# Letter to the Editor: Licensure Portability for Occupational Audiologists is Essential

**DOI:** 10.5195/ijt.2017.6234

**Published:** 2017-11-20

**Authors:** GEORGE R. COOK

**Affiliations:** WORKPLACE INTEGRA, INC., GREENSBORO, NC

**Keywords:** Licensure, Occupational audiologists, Telehealth, Telepractice

## Abstract

Occupational audiologists have a crisis in their profession and need advocates. These audiologists are primarily responsible for industrial hearing conservation programs and their compliance with multiple regulations, such as Occupational Safety and Health Administration (OSHA), Mine Safety and Health Administration (MSHA) and the Federal Railroad Administration. Occupational hearing programs, for the most part, are multi-state programs as companies and corporations are national organizations. Also, companies may contract services across state lines as local services may not be desired or available. Individual state telepractice regulations require audiologists who are professionally supervising these programs via the internet and phone, to secure licensure in each state. For this licensure redundancy, the cost in time and tracking are enormous. It is imperative that the American Speech-Language-Hearing Association (ASHA), secure multistate licensure for speech-language pathologists and audiologists. For the profession of occupational audiology, it is essential.

Occupational audiologists are experiencing a crisis in their profession and need advocates. These audiologists are primarily responsible for industrial hearing conservation programs and their compliance with the regulations of agencies such as Occupational Safety and Health Administration (OSHA), Mine Safety and Health Administration (MSHA), and the Federal Railroad Administration (FRA). Their surveillance includes the professional oversight of audiometric monitoring programs, and includes: (1) the review of audiometric tests to stop hearing loss due to occupational hazards, (2) recommendations of audiological medical referrals, and (3) determination of work relatedness for purposes of recording on the OSHA 300 Log.

Individual state telepractice regulations require audiologists who are professionally supervising these programs via the internet and phone to secure licensure in each state. Occupational hearing programs, for the most part, are multi-state programs since companies and corporations are national organizations. Also, companies may contract services across state lines as local services may not be desired or available.

In the service company I am most familiar with, Workplace Integra, Inc., the Hearing Conservation Division employs five audiologists -- two full time and three part-time. The principal audiologist reviewing audiograms is currently licensed in 25 different states due to the locations of our clients. For Workplace Integra to have depth in staffing, the company requires at least two audiologists to become licensed in each of the states it serves. Therefore, staff audiologists must obtain multiple licensures. For example, at least two other staff audiologists have more than 18 state licensures each, and are scheduled for more.

The first difficulty is having several state licensures and adding another. Each state requires upon initial licensure, a Letter of Good Standing. Most states charge from $5.00 to $25.00 per letter. If an audiologist is licensed in 25 states they would need to send out 25 letters requesting that a Letter of Good Standing be sent to the state where a new license is being obtained. To complicate this further, many states have their own forms or letters that must be sent to each state for completion.

Second, licensure renewal is mostly done annually with renewal periods from: the (1) calendar year, (2) birthday, (3) date of original licensure, or (4) arbitrarily (e.g., July 1 to June 30). A few states have renewal dates of two years (e.g., Oklahoma) and one state (i.e., New York) requires renewal every three years. Renewal fees can range from $60 to over $200 for each state.

Third, each state has different requirements for Continuing Education Units. Anywhere from 10 to 40 CEUs may be required over one, two, or three year periods. The states’ renewal dates do not always coincide. Some states require 2 or 3 of these CEUs to be in the area of Ethics.

Fourth, though a few of the above clerical tasks can be delegated to support staff, the audiologist holds the ultimate responsibility for obtaining and renewing a state license, and meeting the CEU requirements of quantity and topic. These burdensome tasks are a major threat to our profession.

Currently, more than a dozen states require that the audiologist hold an AuD degree. It seems safe to assume that in the future all states will have this requirement. What doctoral level professional capable of contributing significantly to the hearing preservation of the American worker is going to want to spend 10% to 20% of his or her time obtaining, renewing, and qualifying for licensure in 20, 30, or more individual states? These time demands are in addition to the inadvertent consequences of engaging in telepractice in a state without a license, clients who process a hearing test done in another state, or miscalculating a renewal date. Discipline in one state would result in discipline in several states.

For this licensure redundancy, the cost in time and tracking are enormous. For example, an audiologist with 30 state licenses would have between $3,000 and $3,500 in annual fees. To add an additional state could cost more than $400 in fees and postage. All this is in addition to the non-productive hours spent tracking and renewing licenses by mail and on the internet. If we multiply the time and money it costs to have multiple staff audiologists licensed, the resulting cost is immense.

If we do not have multiple state licensure for the occupational audiologists, and the profession cannot therefore meet the need for professional supervision of these audiometric programs, government and industry will empower lesser qualified professions to provide program supervision (e.g., nurses, physician assistants, technicians, etc.).

It is imperative for our national association, the American Speech-Language-Hearing Association (ASHA), to secure multistate licensure for speech-language pathologists and audiologists. For the profession of occupational audiology, it is essential.
